# Hyperlipidemia in immune thrombocytopenia: a retrospective study

**DOI:** 10.1186/s12959-023-00545-9

**Published:** 2023-10-02

**Authors:** Shouqing Han, Hui Lu, Yafei Yu, Xinguang Liu, Fangmiao Jing, Liang Wang, Yajing Zhao, Ming Hou

**Affiliations:** 1 Department of Hematology, Qilu Hospital of Shandong University, 107 West Wenhua Road, Jinan, 250012 Shandong China; 2 Shandong Provincial Key Laboratory of Immunohematology, Qilu Hospital of Shandong University, Jinan, China; 3 Affiliated Hospital of Weifang Medical University, Weifang, Shandong China; 4 Department of Geriatric Medicine, Qilu Hospital of Shandong University, Jinan, China; 5 Department of Hematology, Shengli Oilfield Central Hospital, Dongying, China; 6 Leading Research Group of Scientific Innovation, Department of Science and Technology of Shandong Province, Qilu Hospital of Shandong University, Jinan, China

**Keywords:** Immune thrombocytopenia, Bleeding, Lipid metabolism, Statins

## Abstract

**Background:**

Immune thrombocytopenia (ITP) is an autoimmune hemorrhagic disease characterized by low platelet count and bleeding manifestations. However, some patients also suffered from atherosclerosis or even infarction. Apart from activated platelets, lipid metabolism takes a large part in the formation of atherosclerosis and metabolic syndrome. The lipid metabolic state in ITP patients is still unknown.

**Methods:**

We retrospectively reviewed 302 hospitalized ITP patients in our cohort, comparing their blood lipids, bleeding symptoms, metabolic diseases and treatment responses.

**Results:**

We found a high proportion of ITP patients suffered from hyperlipidemia, and other metabolic diseases including cardiovascular or cerebral atherosclerosis or infarction, hypertension, and type 2 diabetes. Hyperlipidemia was associated with severe bleeding and treatment refractoriness in ITP. Statins could alleviate thrombocytopenia and bleeding severity, and facilitate ITP treatment, while improving hyperlipidemia in ITP patients.

**Conclusions:**

Our present study demonstrated that lipid metabolism might play an indispensable role in ITP pathogenesis and development.

**Supplementary Information:**

The online version contains supplementary material available at 10.1186/s12959-023-00545-9.

## Introduction

Treating patients with both thrombocytopenia and cardiovascular or cerebrovascular thrombosis poses a dilemma in clinical practice. However, this condition is common in patients with immune thrombocytopenia (ITP) [1–3]. ITP is the most common autoimmune hemorrhagic disorder characterized by platelet deficiency [4]. Specifically, the platelet count is not completely related to bleeding severity in patients with ITP [5, 6]. Some patients seldom have severe bleeding, whereas others may exhibit both severe bleeding and venous or arterial thrombosis despite low platelet count [3]. Patients with ITP have an increased risk of thrombosis, but studies with strictly matched controls are needed to confirm these findings [7]. Thrombus formation tendency existing with a low platelet count seems paradoxical, and its mechanism is yet unclear. Studies have identified several contributing factors to thrombosis in ITP, such as age, metabolic syndrome, smoking, the proinflammatory state, higher number of reticulated platelets, presence of antiphospholipid antibodies, and ITP treatments including intravenous immunoglobulin, corticosteroids, thrombopoietin-receptor agonists (TPO-RAs), and splenectomy [2, 3, 8–10].

Several studies have demonstrated that atherosclerosis and hyperlipidemia are more common in patients with autoimmune diseases, such as psoriasis, systemic lupus erythematosus (SLE), and rheumatoid arthritis (RA), than in the general population [11–13]. Dysregulation in lipid metabolism influences the pathogenesis of autoimmune diseases. Moreover, autoimmunity and chronic inflammation also play pivotal roles in metabolic syndrome [11, 14, 15]. Statins are commonly used in the treatment of hyperlipidemia and atherosclerosis through competitive combination with 3-hydroxy-3-methyl-glutaryl-co-enzyme A reductase [16]. Intriguingly, statins have immunomodulatory and anti-inflammatory effects on a variety of immune cells, such as T cells and antigen-presenting cells, which could be used in the treatment of autoimmune diseases [17, 18].

Atorvastatin is involved in the regulation of ITP via the modulation of bone marrow endothelial cell function and T cell immunity [19, 20]. However, the lipid profile in ITP is yet to be investigated. It is unclear whether patients with ITP are prone to impaired lipid metabolism contributing to atherosclerosis. Moreover, it is unclear whether the potential therapeutic effect of statins is exerted partly by the alteration of lipid metabolism in ITP.

Here, we conducted a retrospective study to evaluate pre-existing clinical metabolic comorbidities in patients with ITP, and investigate the impact of lipid metabolism on the treatment and prognosis of ITP.

## Methods

### Patients and controls

Totally 302 patients with active ITP (126 men and 176 women; 18–92 years old; median age, 53 years) were enrolled between October 2016 and September 2018 at the Department of Hematology, Qilu Hospital of Shandong University, China, and Department of Hematology, Shengli Oilfield Central Hospital, Dongying, China. The duration of ITP ranged from 1 day to > 30 years. All patients fulfilled the diagnostic criteria for ITP recommended by the 2011 American Society of Hematology guidelines [21]. Patients with secondary ITP and pregnant women were excluded. A total of 105 age- and sex-matched controls (49 men and 56 women; 24–86 years old; median age, 45 years) were enrolled from the physical examination center of Qilu Hospital. Patients with hematological and autoimmune diseases, and pregnant women were excluded from the control group. Our study was approved by the ethics committees of the two participating hospitals.

### Data collection

Peripheral blood samples were obtained from patients with ITP and controls for blood tests. Lipid levels, including low-density lipoprotein cholesterol (LDL-C), high-density lipoprotein cholesterol (HDL-C), total cholesterol (TC), and triglyceride (TG), and platelet counts, were collected on the first day of hospitalization. The normal range is 0.30–1.70 mmol/L for TG, 1.00-3.37 for LDL-C, 2.80-6.00 for TC, and 0.80-2.00 for HDL-C. Hyperlipidemia is defined as LDL-C, TC, or TG above the upper limit of the normal range, or HDL-C below the lower limit of the normal range in the present study. Moreover, bleeding scores, body mass index (BMI), pre-existing clinical comorbidities, and treatments were recorded. Bleeding scores were graded using the ITP Bleeding Scale (IBLS) system by evaluating the severity of skin, mucous, and organ bleeding [22]. The metabolic diseases in the present analysis included pre-existing cardiovascular or cerebrovascular atherosclerosis or infarction, hypertension, and type 2 diabetes mellitus (T2DM) before ITP identification or treatment. Patients with T2DM and hypertension secondary to corticosteroid treatment were excluded. The criteria for treatment responsiveness (R) included a platelet count of over 30 × 10^9^ /L and at least a two-fold growth from baseline levels without bleeding symptoms after at least a one-month follow-up. Otherwise, the treatment was considered failed and was regarded as having no response (NR) [23].

### Propensity score-matched method

The propensity score matching (PSM) method was used to balance confounding factors, including age and sex, while comparing patients with ITP with or without a history of statin use [24]. The matching ratio was 1:7 for patients with ITP who had previously used statins versus those who had not. PSM was also used to balance the previous corticosteroid treatment when comparing characteristics of patients with hyperlipidemia and normal blood lipid profile. Exact matching with a caliper size of 0.4 was conducted for matching pairs according to the propensity scores. The balance was assessed by evaluating standardized differences (SD) before and after matching. Only those with an SD smaller than 0.1 were recognized as qualified balancing.

### Statistical analysis

Data are expressed as medians with interquartile ranges or means ± SEM. Mann-Whitney test, Student t-test, chi-squared test, One-way ANOVA analysis with Tukey’s multiple comparisons, and logistic regression analysis were used to analyze the results. *P*-values < 0.05 were considered statistically significant. Statistical analysis was performed using the SPSS statistical software (version 19) and GraphPad Prism software (version 7).

## Results

### Impaired blood lipid profile in patients with ITP

**Fig. 1:**
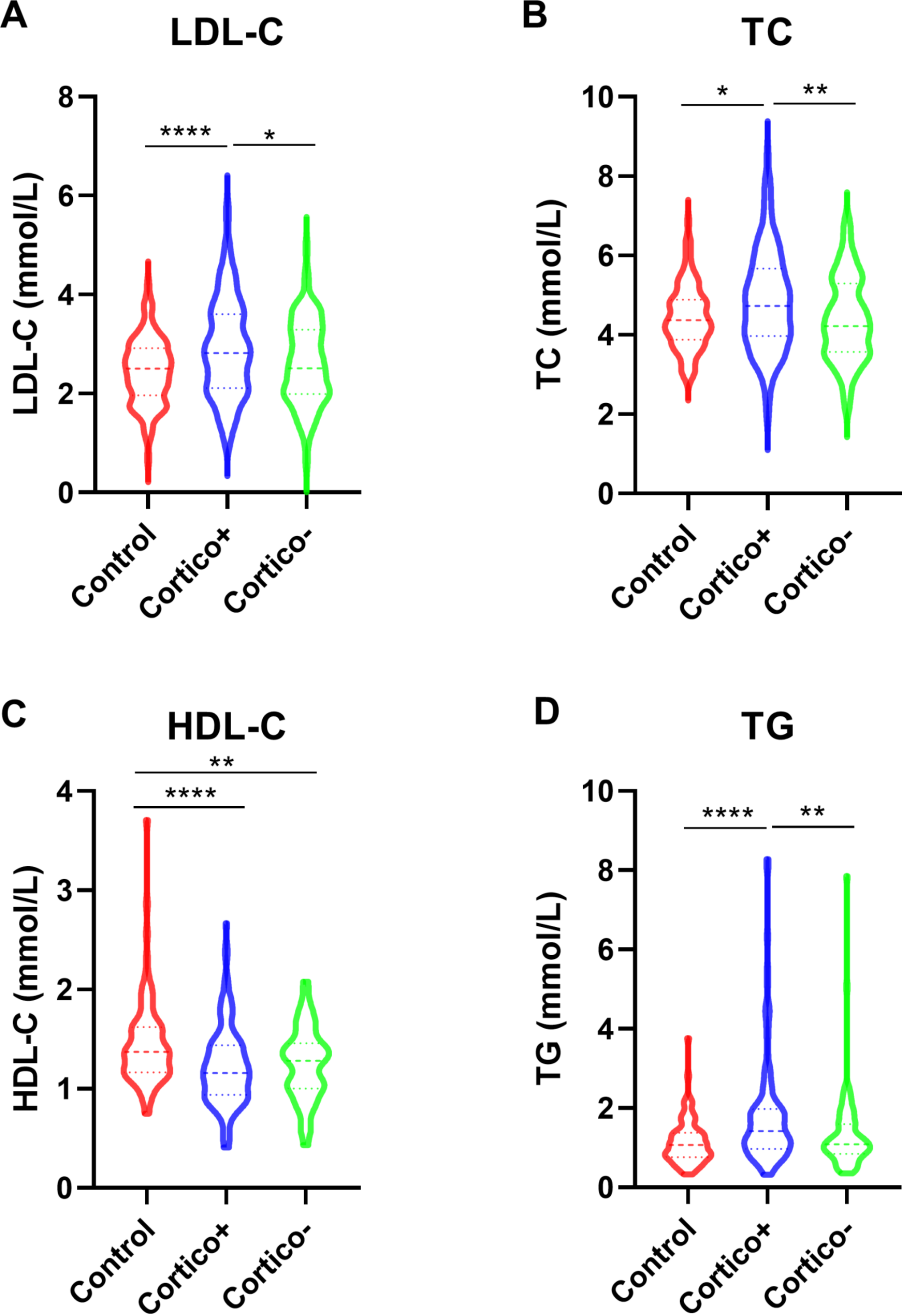
Blood lipid profile of ITP patients and controls. Violin plot of subgroup analysis of blood lipid profile among controls, ITP patients with or without previous corticosteroid treatment, including LDL-C (**A**), total cholesterol (**B**), HDL-C (**C**), and triglyceride (**D**). *: P < 0.05, **: P < 0.01, ****: P < 0.0001

Lipid levels were compared between patients with ITP and controls. Levels of LDL-C and TG were apparently higher in patients with ITP (*P* = 0.003 and *P* = 0.0012, respectively), and the HDL-C levels were lower than those in controls (*P* < 0.0001, Table 1). Over half (50.7%) of the patients with ITP had abnormal lipid levels, and 39.7% had pre-existing metabolic diseases before ITP identification or treatment, including cardiovascular or cerebrovascular atherosclerosis or infarction (16.2%), hypertension (25.8%), and T2DM (19.2%). The WBC and neutrophil counts were significantly higher in patients with ITP than those in controls, which is in accordance with the previously published results [3, 7]. The hemoglobin levels were lower in ITP patients compared to controls. It is probably due to different degrees of bleeding in ITP patients (Table 1). Since corticosteroid therapy of ITP patients confounds with increasing blood lipid levels, subgroup analysis was employed to compare the lipid profile of ITP patients with/without previous corticosteroid treatment to that of controls (Fig. 1). Patients with corticosteroids had higher levels of LDL, cholesterol, and triglyceride compared to controls and patients without corticosteroids, and lower levels of HDL compared to controls (Fig. 1). Interestingly, patients without corticosteroid treatment also exhibited a decreased level of HDL compared to controls (Fig. 1), suggesting the underlying lipid metabolic disorder in ITP patients. As pre-existing metabolic disease might confound with lipid levels of ITP patients, the blood lipid profile of ITP patients without metabolic diseases (n = 234) were also analyzed, and the results were in consistence with total ITP patients (Supplemental Tables 1, and Supplemental Fig. 1).

**Table 1:** Clinical characteristics of ITP patients and controls

	Controls	ITP patients	P
Age (median, interquartile range)	45 (27.5)	53 (26)	0.137
Gender(male %)	49 (46.7%)	126 (41.7%)	0.423
LDL-C (mmol/L) (mean ± SEM)	2.45 ± 0.08	2.74 ± 0.05	0.003**
HDL-C (mmol/L) (mean ± SEM)	1.44 ± 0.05	1.22 ± 0.02	< 0.0001****
TC (mmol/L) (mean ± SEM)	4.44 ± 0.04	4.58 ± 0.07	0.261
TG (mmol/L) (mean ± SEM)	1.13 ± 0.06	1.52 ± 0.07	0.0012**
WBC (x10^9^/L) (mean ± SEM)	6.05 ± 0.19	7.11 ± 0.44	0.0018**
Neu (x10^9^/L) (mean ± SEM)	3.72 ± 0.17	5.01 ± 0.39	0.0009***
HGB (g/L) (mean ± SEM)	140.43 ± 2.27	126.2 ± 4.25	0.002**

LDL-C: low density lipoprotein cholesterol, HDL-C: high density lipoprotein cholesterol, TC: total cholesterol, TG: triglyceride, WBC: white blood cell, Neu: neutrophil, HGB: hemoglobin

**: P < 0.01, ***: P < 0.001, ****: P < 0.0001

The median age of patients with ITP and hyperlipidemia was 54 years, and 42.5% of them were men, which was comparable to that of those with normal lipid levels (Table 2). Furthermore, patients with hyperlipidemia had a longer ITP duration (*P* = 0.03, Table 2). Our study indicated that patients with ITP were prone to metabolic syndrome, including hyperlipidemia, especially in patients with corticosteroid use.

**Table 2:** Clinical characteristics of ITP patients with high lipid levels and normal lipid levels

	High lipid	Normal lipid	P
Age (median, interquartile range)	54 (18.5)	52 (34.5)	0.488
Gender(male %)	65 (42.5%)	62 (41.6%)	0.725
ITP duration (months) (mean ± SEM)	50.5 ± 8.89	27.53 ± 5.59	0.03*
Initial platelet counts (×10^9^/L) (mean ± SEM)	18.31 ± 2.21	19.98 ± 1.880	0.57
Platelet counts after treatment (×10^9^/L) (mean ± SEM)	74.16 ± 5.30	98.27 ± 6.87	0.0057**
Severe bleeding (n, %)	51 (33.3%)	22 (14.8%)	0.0001***
Corticosteroid treatment (R, %)	107 (81.1%)	99 (85.34%)	0.4
TPO agent treatment (R, %)	53 (58.9%)	49 (75.4%)	0.04*
Refractory (n, %)	25 (16.3%)	4 (2.68%)	0.0001***

*: P < 0.05, **: P < 0.01, ***: P < 0.001

### Hyperlipidemia were correlated with severe bleeding in ITP

Patients with ITP and hyperlipidemia had lower platelet counts after treatment (*P* = 0.0057) and more severe bleeding symptoms than patients with normal lipid levels (*P* = 0.0001, Table 2). Propensity score matching (PSM) was utilized to balance the previous administration of corticosteroid therapy between patients with hyperlipidemia and normal blood lipid profile, including the duration, the type (dexamethasone or prednisone), and the withdraw time of corticosteroids. After matching, ITP patients with hyperlipidemia still had lower platelet count after treatments (*P* = 0.08), and more severe bleeding symptoms compared to patients with normal lipid levels (*P* = 0.005, Supplemental Table 2). Furthermore, we investigated the factors influencing bleeding severity using univariate (Supplemental Table 3) and multivariate logistic regression analyses (Table 3), including age, sex, hyperlipidemia, metabolic diseases, BMI, platelet counts, and previous corticosteroid treatment. Hyperlipidemia was an independent influencing factor for bleeding severity in patients with ITP (*P* = 0.0001, Table 3). However, the subgroups of lipids (LDL-C, HDL-C, TG, and TC) ,the specific pre-existing metabolic diseases (cardiovascular or cerebrovascular atherosclerosis or infarction, hypertension, and T2DM), and previous use of corticosteroid treatment were not found to be individually correlated with bleeding severity (Supplemental Table 3).

**Table 3:** Multivariate analysis for bleeding severity in ITP patients

	OR	95% CI		*P* value
		Lower	Upper	
Age	1.133	0.648	1.980	0.661
Gender	0.987	0.969	1.005	0.160
Hyperlipidemia	3.004	1.687	5.351	0.0001***
Metabolic diseases#	1.311	0.694	2.477	0.404
BMI	0.983	0.919	1.051	0.615
Platelet	0.994	0.981	1.006	0.319

#Metabolic diseases: including pre-existed cardiovascular or cerebrovascular atherosclerosis or infarction, hypertension, and type 2diabetes mellitus before ITP identification or treatments

***: P < 0.001

### Patients with metabolic diseases responded better to corticosteroids

Most patients enrolled in our study received first- or second-line treatment, among which 38.4% were newly diagnosed with ITP. Other patients who had received treatment elsewhere were recorded for treatment effects by reviewing their medical histories. In total, 248 patients received corticosteroid treatment. The initial response rate to corticosteroids in our study was 83.1%, which is in agreement with the results of previous studies [25]. We evaluated the factors influencing the responsiveness to corticosteroids. The univariate analysis indicated that patients with high LDL-C levels tended to be refractory to corticosteroid treatment (*P* = 0.015; Supplemental Table 4). Thus, we included LDL levels in the following multivariate analysis. However, the LDL-C levels were not found to have significant influence on corticosteroid treatment (Supplemental Table 5). And the HDL-C, TC, and TG levels also had no influence on corticosteroid treatment. Interestingly, patients with other metabolic diseases, specifically hypertension and T2DM, tended to have a better response to corticosteroids (*P* = 0.048 and *P* = 0.021, respectively; Supplemental Table 4). Further evaluation using multivariate logistic regression analysis showed that patients with metabolic diseases tended to have a higher corticosteroid response rate than those without metabolic diseases (91.4% vs. 78.1%, *P* = 0.002, Table 4). Hyperlipidemia and BMI scores did not correlate with corticosteroid response. Additionally, we analyzed the influencing factors associated with corticosteroid responsiveness in patients without previous corticosteroid treatment (n = 112). However, no significant influencing factors were found, except for gender, which might be due to a limited number of the patients (Supplemental Table 6).

**Table 4:** Multivariate analysis for corticosteroid treatment in ITP patients

	OR	95% CI		*P* value
		Lower	Upper	
Age	1.019	0.997	1.042	0.096
Gender	1.878	0.925	3.812	0.081
Hyperlipidemia	1.289	0.621	2.674	0.495
Metabolic diseases	0.251	0.102	0.614	0.002**
BMI	1.052	0.967	1.144	0.238
Platelet	0.971	0.941	1.001	0.061

**: P < 0.01

### BMI scores were associated with TPO-agent treatment effects

Patients who were refractory or remissive to corticosteroids were administrated secondary treatment. TPO agents are the most considered choice for second-line treatments. Totally 156 patients received TPO agents, including recombined human TPO and eltrombopag (eltrombopag was administered at an initial dose of 25 mg and adjusted according to the platelet response to a maximum of 75 mg according to the dietary rules). Patients with hyperlipidemia had worse TPO agent reactivity than those with normal lipid levels (58.9% vs. 75.4%, *P* = 0.04, Table 2, and 39.5% vs. 69.5%, *P* = 0.04 after PSM, Supplemental Table 2). We analyzed the factors related to responsiveness to TPO agents. In the univariate analysis, hyperlipidemia and high BMI scores were correlated with poor TPO-agent response (*P* = 0.029 and *P* = 0.002, respectively; Supplemental Table 7). Interestingly, patients with T2DM tended to respond better to TPO agents (*P* = 0.021, Supplemental Table 7). The multivariate logistic regression analysis indicated that BMI scores were independently associated with the effect of TPO-agent treatment (*P* = 0.004, Table 5).

**Table 5:** Multivariate analysis for TPO-agent treatment in ITP patients

	OR	95% CI		*P* value
		Lower	Upper	
Age	1.003	0.979	1.026	0.831
Gender	1.308	0.640	2.670	0.462
Hyperlipidemia	1.838	0.867	3.898	0.112
Metabolic diseases	0.654	0.275	1.554	0.336
BMI	1.132	1.040	1.233	0.004**
Platelet	0.984	0.963	1.006	0.162

**: P < 0.01

### More patients with hyperlipidemia were refractory to ITP treatment

Patients who failed the initial treatments and were resistant to rituximab and TPO agents or splenectomy were regarded as having refractory ITP according to the Chinese ITP guidelines [26]. Through a careful review of the patients’ clinical histories, 29 patients were considered to have refractory ITP in the present study. More patients with hyperlipidemia had refractory ITP compared to those with normal lipid levels (16.3% vs. 2.68%, *P* = 0.0001, Table 2). We further evaluated the risk factors of refractory ITP using univariate (Supplemental Table 8) and multivariate logistic regression analyses (Table 6). Hyperlipidemia and low platelet count were significantly associated with refractory ITP (*P* = 0.001 and *P* = 0.023, respectively; Table 6). Specifically, high TC and TG levels contributed to treatment failure (*P* = 0.020 and *P* = 0.009, respectively; Supplemental Table 8). However, comorbidities with metabolic diseases and BMI scores did not significantly influence the refractoriness of ITP treatment (Table 6). No significant influence of hyperlipidemia was found on other second-line therapies, including decitabine, rituximab, vindesin, splenectomy, danazol, Cyclosporin A, and IL-11 (Supplemental Table 9).

**Table 6:** Multivariate analysis of influencing factors for refractory ITP

	OR	95% CI		*P* value
		Lower	Upper	
Age	1.628	0.710	3.732	0.250
Gender	0.979	0.952	1.006	0.126
Hyperlipidemia	6.957	2.304	21.005	0.001**
Metabolic diseases	1.368	0.535	3.499	0.514
BMI	0.980	0.886	1.085	0.698
Platelet	0.950	0.908	0.993	0.023*

*: P < 0.05, **: P < 0.01

### The use of statins could benefit ITP treatment

Among patients with hyperlipidemia, only 18 patients were administrated with statins, and most of them were previously diagnosed with coronary or cerebral atherosclerosis before ITP diagnosis or treatment, and were using statins to the data collection. The duration of statin treatment ranged from 1 to 10 years, with a median time of 4 years. The median age of patients treated with statins was 62.5 years, which was significantly higher than that of those without statin treatment (53 years, *P* = 0.003). The gender distribution did not differ. We further analyzed the effects of statins after PSM for age and sex. After PSM, 17 patients used statins and 86 patients did not; these patients were comparable in age, sex, and ITP duration. Patients with statins had lower levels of LDL-C (*P* = 0.0394) and TC (*P* = 0.073), indicating that statins exerted regulatory effects on lipid metabolism. Intriguingly, the initial platelet counts before ITP treatment were markedly higher in patients taking statins than in those not taking statins (*P* = 0.0022). Furthermore, statin treatment ameliorated bleeding severity (*P* = 0.02), indicating that using statins might alleviate ITP severity. However, statin administration did not influence platelet count after ITP treatment. We further investigated the effects of statins on ITP treatment. It was shown that, compared to patients without statins, all patients with statins were responsive to corticosteroids (100% vs. 66.2%, *P* = 0.016), and none of them had refractory ITP (0% vs. 18.5%, *P* = 0.066), indicating that statins might facilitate ITP treatment, especially corticosteroids. However, statins did not improve TPO agent treatment (Table 7).

**Table 7:** Therapy and outcome of ITP with hyperlipidemia stratified by statin therapy

	With statin treatment (n = 17)	Without statin treatment (n = 86)	*P* value
Age (years) (median, interquartile range)	62 (16)	58 (10.75)	0.0651
Gender(male %)	7 (41.2%)	31 (36.0%)	0.692
ITP duration (months) (mean ± SEM)	46.53 ± 21.69	51.91 ± 8.93	0.804
Initial platelet counts (×10^9^/L) (mean ± SEM)	42.18 ± 14.7	16.90 ± 2.04	0.0022**
Platelet counts after treatment (×10^9^/L) (mean ± SEM)	81.36 ± 12.29	74.59 ± 7.44	0.72
LDL-C (mmol/L) (mean ± SEM)	2.75 ± 0.25	3.30 ± 0.10	0.0394*
HDL-C (mmol/L) (mean ± SEM)	1.04 ± 0.08	1.23 ± 0.04	0.076
TC (mmol/L) (mean ± SEM)	4.65 ± 0.27	5.22 ± 0.13	0.073
TG (mmol/L) (mean ± SEM)	2.36 ± 0.35	1.92 ± 0.14	0.23
Severe bleeding (n, %)	0 (0%)	30 (34.9%)	0.02*
Corticosteroid treatment (R, %)	12 (100.0%)	49 (66.2%)	0.016*
TPO agent treatment (R, %)	6 (85.7%)	28 (50.9%)	0.116
Refractory (n, %)	0 (0%)	14 (16.3%)	0.066

*: P < 0.05, **: P < 0.01

## Discussion

A low platelet count and bleeding are common clinical manifestations in ITP [22]. However, some patients with ITP have thrombotic comorbidities. Oxidized LDL-C is pivotal in the development of atherosclerosis as it enters the intima of arteries and represents a crucial pro-inflammatory stimulus [27]. The lipid metabolic state and comorbidities related to atherosclerosis in patients with ITP remain to be elucidated. Our study established the first cohort to determine metabolic comorbidities and lipid metabolism profiles in ITP.

Lipid metabolism involves the production of lipid species (cholesterol, fatty acids, and phospholipids) and the breakdown of lipid species via fatty acid oxidation. In metabolic disorders such as obesity and atherosclerosis, various classes of lipids interact with immune cells and affect innate and adaptive immune responses, which in turn promote autoimmune and inflammatory activities [28, 29]. Recent advances have revealed that pathways promoting lipid synthesis and accumulation tend to drive a pro-inflammatory phenotype of macrophages and T lymphocytes, whereas pathways enhancing β-oxidation and lipid efflux lead to the anti-inflammatory phenotype [30–32]. Furthermore, hyperlipidemia is associated with increased platelet activation and promotes atherosclerosis formation. Statins have pleiotropic effects including the stabilization of arterial plaques, normalization of endothelial functions, and reduction of platelet activation and subclinical inflammation [33–35]. Here, we demonstrated a high proportion of hyperlipidemia in patients with ITP, as well as metabolic diseases including atherosclerosis, hypertension, and T2DM, which might explain the high incidence of atherosclerosis in patients with ITP despite a low platelet count. Corticosteroids are the most frequently used therapies in ITP patients, and contribute to metabolic disturbances such as impaired glucose tolerance, metabolic syndrome and hyperlipidemia. It partly explained why so many ITP patients were hyperlipidemic. Interestingly, ITP patients without previous corticosteroid use still have lower HDL levels compared to controls, which is consistent with previous studies that low HDL level is correlated with a high risk of autoimmune disease [36]. Moreover, it has been reported that platelet activation level is higher in ITP patients compared to that in normal controls [37, 38]. In our present study, statins were found to relieve bleeding in ITP, which might be related with improved endothelial and platelet function.

Inflammatory reactions have been identified as a possible cause of bleeding in thrombocytopenia [30, 31]. Our previous study has found that patients with ITP and severe bleeding had high pro-inflammatory cytokine levels, indicating that inflammation could contribute to bleeding risk in ITP [39]. Studies have found the neutrophil count, neutrophil activation, and neutrophil extracellular trap (NET) formation were increased in ITP patients [3, 7]. Platelet–neutrophil interaction mediated by CD62P and PSGL1 could modulate B-cell activating factor (BAFF) production from neutrophils, and promote neutrophil activation and recruitment to inflammatory sites [38]. In the present study, we further identified that patients with hyperlipidemia were prone to more severe bleeding and lower platelet count after treatment, indicating that hyperlipidemia might contribute to bleeding propensity and interfere with platelet recovery, which might be related to enhanced pro-inflammatory reactivity in patients with ITP and hyperlipidemia. Whether hyperlipidemia exacerbates bleeding through modulating platelet-neutrophil interaction in ITP were undefined. Further studies are needed to investigate the role of hyperlipidemia and statins in the process of thrombosis and hemostasis in ITP.

Cholesterol is a fundamental component of lipid raft formation in cell membranes and influences membrane permeability, signaling, and transportation [40]. Glucocorticoid receptor (GR), a cytoplasmic steroid receptor that functions as a ligand-activated transcriptional regulator, is localized in lipid rafts and regulates several GR-dependent cellular responses [41, 42]. Membrane cholesterol was found to inhibit the insertion and trans-nucleation of cortisone into lipid bilayers, suppressing cortisone crystallite formation, one of the steroid-membrane interactions [43]. Therefore, blood lipids may interfere with corticosteroid response. We analyzed the influence of lipid levels on corticosteroid treatment in patients with ITP, and high LDL-C levels were associated with a poor response to corticosteroids, which is probably related to the interaction between lipid rafts and GR signaling transduction. Furthermore, hyperlipidemia and high BMI scores were correlated with poor TPO-agent responses. More patients with hyperlipidemia had refractory ITP, indicating that lipid metabolism might participate in immune regulation during ITP development and progression.

In addition to lipid metabolism, we also analyzed the relationship between other metabolic diseases and ITP bleeding severity and treatment responses, including pre-existing cardiovascular or cerebrovascular atherosclerosis or infarction, hypertension, and T2DM, before ITP identification or treatment. Metabolic disease and BMI scores were not correlated with ITP bleeding severity. Interestingly, patients with previously diagnosed metabolic diseases, especially those with hypertension and T2DM, had better corticosteroid responses. Patients with T2DM also showed a better response to TPO agents. These findings were in agreement with our previously reported study that patients with ITP and T2DM had a better ITP treatment response, which might be a result of administration of metformin [23]; however, these findings were in contrast to those of another study that showed that patients with ITP and hypertension or diabetes had worse purpura and poorer responses to corticosteroids [44]. The influence of metabolic syndrome on ITP might vary with age, disease duration, ethnicity, and treatment regimens, which awaits further evaluation.

Remission in patients with SLE and RA after treatment is associated with an improvement in hyperlipidemia [27, 45]. Statins were also found to improve disease activity through an anti-inflammatory effect by the modification of vascular risk factors in patients with RA [46]. Recent studies have highlighted the possible treatment effect of statins on ITP via the modulation of bone marrow endothelial cell function and T cell immunity [19, 20]. Since bone marrow endothelial cells are essential in megakaryocytopoiesis, whether statins could regulate the differentiation and platelet production of megakaryocytes remains to be further investigated. Here, among patients with hyperlipidemia, those treated with statins had higher initial platelet counts and lower bleeding severity. Moreover, fewer patients treated with statins were refractory to corticosteroids or developed refractory ITP. Therefore, statins may alleviate ITP severity and facilitate treatment. However, among the patients with hyperlipidemia in the present study, only 11.8% were routinely administered statins, and most of them were previously diagnosed with coronary or cerebral atherosclerosis before ITP diagnosis or treatment. Effective blood lipid reduction is recommended for patients with ITP in future clinical practice. Larger cohorts are needed to further evaluate the effects of statins, including the dose, duration, and type of statins on ITP and other thrombocytopenic diseases.

Our study is the first to demonstrate that, in addition to atherosclerosis formation, hyperlipidemia contributes to severe bleeding, and is related to treatment failure in ITP. Statins can alleviate thrombocytopenia and bleeding severity, and facilitate ITP treatment, while improving hyperlipidemia in patients with ITP. Therapies targeting hyperlipidemia, such as statins, might be new prospects for ITP treatment. There are several limitations in our study. Since it is a retrospective study, we cannot draw the causal correlation between ITP and hyperlipidemia, and further studies with follow up are needed. The controls were enrolled from the physical examination center of Qilu Hospital, and they cannot represent the entire normal population. Patients in our study were mostly patients with active ITP; thus, the lipid profile of patients in remission still require further evaluation.

## Conclusions

A high proportion of ITP patients suffered from metabolic disorders, especially hyperlipidemia. Patients with hyperlipidemia may have more severe bleeding symptoms and worse responses to ITP treatments. Statin may help relieve ITP bleeding symptoms and improve ITP treatment.

### Electronic supplementary material

Below is the link to the electronic supplementary material.

Supplementary Material 1

Supplementary Material 2

## Data Availability

The datasets analyzed during the current study are available from the corresponding author on reasonable request.

## List of Abbreviations

ITP: Immune thrombocytopenia
TPO: Thrombopoietin-receptor agonist
SLE: Systemic lupus erythematosus
RA: Rheumatoid arthritis
LDL-C: Low-density lipoprotein cholesterol
HDL-C: High-density lipoprotein cholesterol
TC: Total cholesterol
TG: Triglyceride
BMI: Body mass index
IBLS: ITP Bleeding Scale
T2DM: Type 2 diabetes mellitus
R: Responsiveness
NR: No response
PSM: Propensity score matching
SD: Standardized differences
GR: Glucocorticoid receptor
