# Supplementary figures and images for: Hyperlipidemia in immune thrombocytopenia: a retrospective study
Source: Thromb J. 2023 Oct 2;21:102. doi: 10.1186/s12959-023-00545-9 (PMC10544441; doi:10.1186/s12959-023-00545-9)

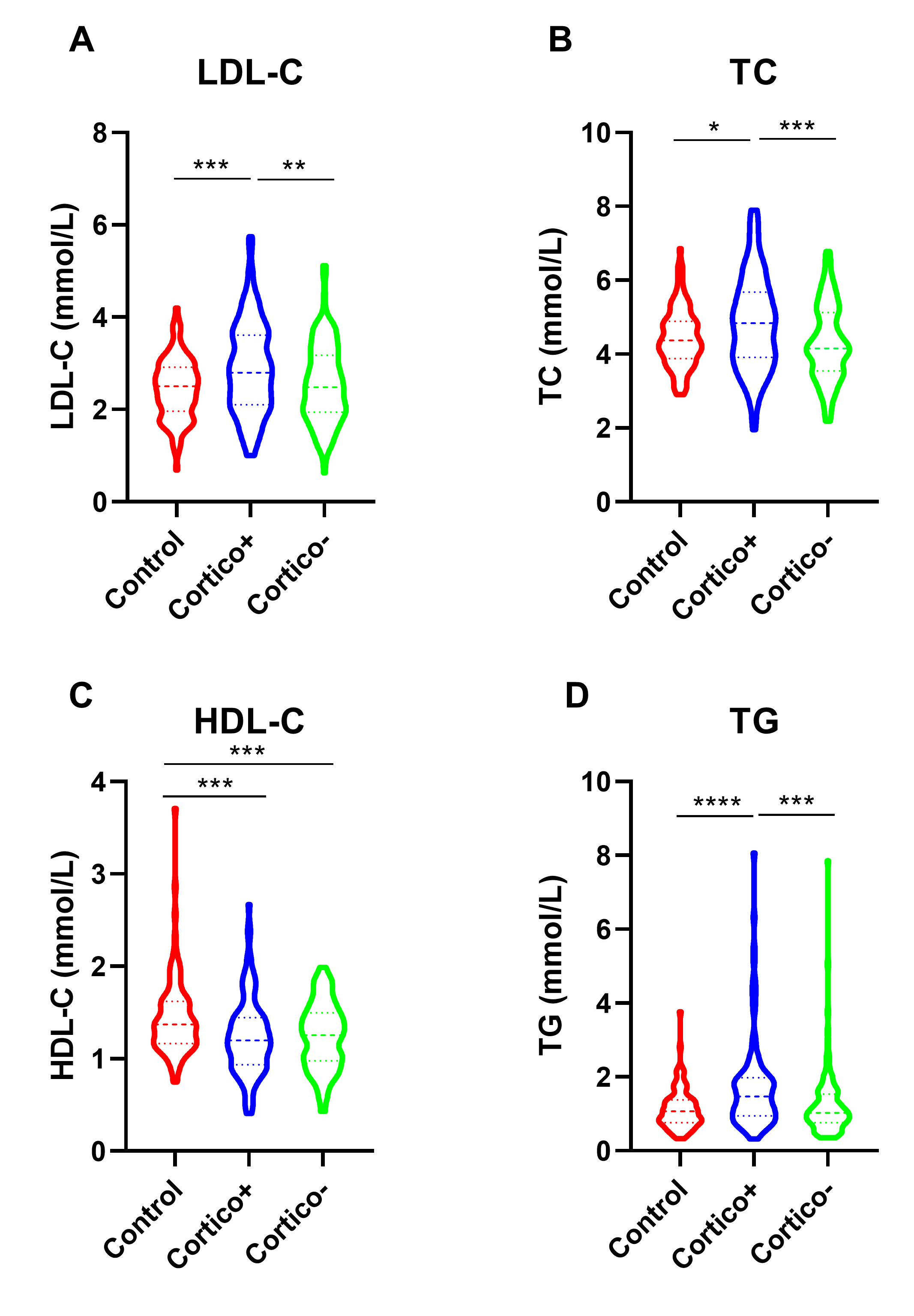

Supplement: Supplementary file 1 — Supplementary Material 1 [file 12959_2023_545_MOESM1_ESM.png]
