# Supplementary material for: Hyperlipidemia in immune thrombocytopenia: a retrospective study
Source: Thromb J. 2023 Oct 2;21:102. doi: 10.1186/s12959-023-00545-9 (PMC10544441; doi:10.1186/s12959-023-00545-9)
Supplement: Supplementary file 2 — Supplementary Material 2 [file 12959_2023_545_MOESM2_ESM.docx]

Supplemental table 1 Clinical characteristics of controls and ITP patients without metabolic diseases

|  | Controls | ITP patients without metabolic diseases | P |
| --- | --- | --- | --- |
| Age (median, interquartile range) | 45 (27.5) | 51 (28.5) | 0.957 |
| Gender（male %） | 49 (46.7%) | 99 (42.3%) | 0.474 |
| LDL-C (mmol/L) (mean ± SEM) | 2.45± 0.08 | 2.73 ± 0.06 | 0.006** |
| HDL-C (mmol/L) (mean ± SEM) | 1.44 ± 0.05 | 1.24 ± 0.03 | < 0.0001**** |
| TC (mmol/L) (mean ± SEM) | 4.44 ± 0.04 | 4.58 ± 0.08 | 0.315 |
| TG (mmol/L) (mean ± SEM) | 1.13 ± 0.06 | 1.48 ± 0.07 | 0.0022** |

LDL-C: low density lipoprotein cholesterol, HDL-C: high density lipoprotein cholesterol, TC: total cholesterol, TG: triglyceride, **: *P* < 0.01, ****: *P* < 0.0001

Supplemental table 2 Clinical characteristics of ITP patients with high lipid levels and normal lipid levels after PSM of corticosteroid use

|  | High lipid | Normal lipid | P |
| --- | --- | --- | --- |
| Age (median, interquartile range) | 54.9 (15.5) | 50.7 (27.5) | 0.16 |
| Gender（male %） | 40 (40%) | 55 (37.2%) | 0.65 |
| ITP duration (months) (mean ± SEM) | 29.60 ± 6.6 | 31.04 ± 5.09 | 0.86 |
| Initial platelet counts (×10^9^/L) (mean ± SEM) | 20.00 ± 2.28 | 19.16 ± 1.92 | 0.78 |
| Platelet counts after treatment (×10^9^/L) (mean ± SEM) | 81.84 ± 7.11 | 99.01 ± 6.60 | 0.08 |
| Severe bleeding (n, %) | 36 (36.0%) | 26 (17.6%) | 0.005** |
| Corticosteroid treatment (R, %) | 49 (80.3%) | 73 (76.0%) | 0.321 |
| TPO agent treatment (R, %) | 17 (39.5%) | 37 (68.5%) | 0.004** |
| Refractory (n, %) | 9 (9%) | 6 (4.05%) | 0.109 |

**: *P* < 0.01

Supplemental table 3 Univariate analysis of influencing factors of bleeding severity in ITP patients

|  | OR | 95% CI | | *P* value |
| --- | --- | --- | --- | --- |
|  |  | Lower | Upper |  |
| Age | 0.993 | 0.978 | 1.008 | 0.34 |
| Gender | 1.121 | 0.658 | 1.908 | 0.674 |
| Hyperlipidemia | 2.886 | 1.643 | 5.072 | 0.001** |
| LDL-C | 1.228 | 0.926 | 1.629 | 0.154 |
| HDL-C | 0.672 | 0.320 | 1.410 | 0.293 |
| TG | 1.197 | 0.958 | 1.495 | 0.113 |
| TC | 1.181 | 0.961 | 1.451 | 0.115 |
| Metabolic diseases# | 1.056 | 0.616 | 1.811 | 0.843 |
| Atherosclerosis† | 0.773 | 0.365 | 1.639 | 0.502 |
| Hypertension | 0.922 | 0.502 | 1.694 | 0.793 |
| T2DM‡ | 0.998 | 0.511 | 1.948 | 0.995 |
| BMI | 1.012 | 0.956 | 1.072 | 0.673 |
| Platelet count | 0.993 | 0.980 | 1.006 | 0.281 |
| Corticosteroid treatment | 1.667 | 0.983 | 2.828 | 0.058 |
| type | 1.646 | 0.913 | 2.961 | 0.096 |
| Duration | 0.986 | 0.971 | 1.001 | 0.075 |
| Withdraw time | 1.002 | 0.986 | 1.019 | 0.784 |

LDL-C: low density lipoprotein cholesterol, HDL-C: high density lipoprotein cholesterol, TC: total cholesterol, TG: triglyceride

#Metabolic diseases: including pre-existed †cardiovascular or cerebrovascular atherosclerosis or infarction, hypertension, and ‡type 2diabetes mellitus before ITP identification or treatments.

**: *P* < 0.01

Supplemental table 4 Univariate analysis of corticosteroid treatment of ITP patients

|  | OR | 95% CI | | *P* value |
| --- | --- | --- | --- | --- |
|  |  | Lower | Upper |  |
| Age | 1.003 | 0.983 | 1.023 | 0.786 |
| Gender | 1.575 | 0.809 | 3.067 | 0.182 |
| Hyperlipidemia | 1.361 | 0.693 | 2.670 | 0.371 |
| LDL-C | 1.568 | 1.092 | 2.250 | 0.015* |
| HDL-C | 0.828 | 0.336 | 2.044 | 0.682 |
| TC | 1.304 | 0.982 | 1.731 | 0.067 |
| TG | 1.190 | 0.948 | 1.493 | 0.134 |
| Metabolic diseases | 0.335 | 0.148 | 0.759 | 0.009** |
| Atherosclerosis | 0.272 | 0.063 | 1.182 | 0.082 |
| Hypertension | 0.371 | 0.139 | 0.992 | 0.048* |
| DM | 0.179 | 0.042 | 0.769 | 0.021* |
| BMI | 1.053 | 0.973 | 1.140 | 0.201 |
| Platelet count | 0.974 | 0.946 | 1.002 | 0.07 |

*: *P* < 0.05, **: *P* < 0.01

Supplemental table 5 Multivariate analysis of corticosteroid treatment of ITP patients

|  | OR | 95% CI | | *P* value |
| --- | --- | --- | --- | --- |
|  |  | Lower | Upper |  |
| Age | 1.017 | 0.994 | 1.040 | 0.155 |
| Gender | 1.975 | 0.966 | 4.039 | 0.062 |
| LDL-C | 1.448 | 0.981 | 2.138 | 0.062 |
| Metabolic diseases | 0.270 | 0.110 | 0.663 | 0.004** |
| BMI | 1.041 | 0.957 | 1.134 | 0.350 |
| Platelet | .970 | 0.939 | 1.001 | 0.058 |

**: *P* < 0.01

Supplemental table 6 Univariate analysis of corticosteroid treatment of ITP patients without previous corticosteroid treatment

|  | OR | 95% CI | | P value |
| --- | --- | --- | --- | --- |
|  |  | Lower | Upper |  |
| Age | 1.001 | 0.975 | 1.028 | 0.914 |
| Gender | 6.053 | 2.295 | 15.966 | 0.0001*** |
| Hyperlipidemia | 0.796 | 0.650 | 0.297 | 2.130 |
| LDL-C | 1.240 | 0.718 | 2.139 | 0.440 |
| HDL-C | 0.424 | 0.125 | 1.434 | 0.167 |
| TC | 1.166 | 0.764 | 1.780 | 0.443 |
| TG | 0.963 | 0.830 | 1.117 | 0.616 |
| Metabolic diseases | 0.564 | 0.191 | 1.665 | 0.300 |
| BMI | 1.004 | 0.943 | 0.894 | 1.128 |
| Platelet count | 1.001 | 0.983 | 1.108 | 0.938 |

***: *P* < 0.001

Supplemental table 7 Univariate analysis of TPO-agent treatment of ITP patients

|  | OR | 95% CI | | *P* value |
| --- | --- | --- | --- | --- |
|  |  | Lower | Upper |  |
| Age | 0.995 | 0.977 | 1.014 | 0.622 |
| Gender | 1.399 | 0.718 | 2.724 | 0.324 |
| Hyperlipidemia | 2.182 | 1.081 | 4.403 | 0.029* |
| LDL-C | 1.302 | 0.928 | 1.828 | 0.127 |
| HDL-C | 0.570 | 0.235 | 1.381 | 0.213 |
| TC | 1.210 | 0.935 | 1.566 | 0.148 |
| TG | 1.181 | 0.918 | 1.520 | 0.197 |
| Metabolic diseases | 0.597 | 0.292 | 1.222 | 0.158 |
| Atherosclerosis | 0.361 | 0.116 | 1.122 | 0.078 |
| Hypertension | 0.712 | 0.329 | 1.540 | 0.388 |
| DM | 0.269 | 0.088 | 0.821 | 0.021* |
| BMI | 1.141 | 1.051 | 1.239 | 0.002** |
| Platelet count | 0.988 | 0.968 | 1.008 | 0.221 |

*: *P* < 0.05, **: *P* < 0.01

Supplemental table 8 Univariate analysis of influencing factors for refractory ITP

|  | OR | 95% CI | | *P* value |
| --- | --- | --- | --- | --- |
|  |  | Lower | Upper |  |
| Age | 0.985 | 0.963 | 1.006 | 0.165 |
| Gender | 1.564 | 0.726 | 3.368 | 0.253 |
| Hyperlipidemia | 7.080 | 2.400 | 20.888 | 0.001** |
| LDL | 1.296 | 0.865 | 1.940 | 0.208 |
| HDL | 0.931 | 0.322 | 2.687 | 0.895 |
| TC | 1.461 | 1.061 | 2.011 | 0.020* |
| TG | 1.372 | 1.082 | 1.739 | 0.009** |
| Metabolic diseases | 0.963 | 0.438 | 2.118 | 0.925 |
| Atherosclerosis | 0.167 | 0.022 | 1.261 | 0.083 |
| Hypertension | 1.105 | 0.468 | 2.607 | 0.820 |
| DM | 0.649 | 0.217 | 1.943 | 0.440 |
| BMI | 1.011 | 0.930 | 1.098 | 0.804 |
| Platelet | 0.944 | 0.904 | 0.985 | 0.009** |

*: *P* < 0.05, **: *P* < 0.01

Supplemental table 9 Influence of hyperlipidemia on the secondary treatment effects

|  | Number | OR | 95% CI | | *P* value |
| --- | --- | --- | --- | --- | --- |
|  |  |  | Lower | Upper |  |
| Decitabine | 70 | 0.667 | 0.249 | 1.787 | 0.420 |
| Rituximab | 35 | 0.367 | 0.075 | 1.797 | 0.216 |
| VDS | 10 | 0.375 | 0.022 | 6.348 | 0.497 |
| Splenectomy | 11 | 3.333 | 0.204 | 54.532 | 0.398 |
| Danazol | 24 | 0.500 | 0.096 | 2.602 | 0.410 |
| CsA | 19 | 1.389 | 0.216 | 8.916 | 0.729 |
| IL-11 | 6 | 1.000 | - | - | 1.000 |
